# Treatment of Acne Vulgaris With a Topical Application of Retinaldehyde, Glycolic Acid, and Silybum marianum Fruit Extract: A Pilot Study

**DOI:** 10.7759/cureus.89218

**Published:** 2025-08-01

**Authors:** Elisete I Crocco, Rossana C Vasconcelos, Christiano S Andrade, Fernanda C Beltrão, Marcus M Santos, Ana L Coutinho

**Affiliations:** 1 Dermatology, Santa Casa de São Paulo School of Medical Sciences, São Paulo, BRA; 2 Dermatology, Nomina Expert Institute, São Paulo, BRA; 3 Dermatology, Universtity of Santo Amaro, São Paulo, BRA; 4 Mathematics, Harven Agribusiness School, São Paulo, BRA; 5 Mathematics, CHR Statistical and Financial Consultancy, São Paulo, BRA; 6 Dermatology, Pierre Fabre Dermocosmetics Brazil, São Paulo, BRA

**Keywords:** acne, acne vulgaris, glycolic acid, retinaldehyde, silybum marianum

## Abstract

Background*:* In acne-prone skin, the loss of homeostasis leads to aberrant epithelial differentiation, promoting the formation of microcomedones. The maintenance of sebaceous stem cell homeostasis has emerged as a new therapeutic target in acne management.

Objective: The aim of this study is to evaluate the efficacy, tolerability, and safety of a topical formulation combining retinaldehyde, glycolic acid, and Silybum marianum fruit extract for the treatment of acne vulgaris.

Methods*:* In this single-arm, open-label, prospective pilot study, eight patients with acne vulgaris were treated with a topical cream containing 7% Silybum marianum fruit extract, 0.1% retinaldehyde, 6% glycolic acid, and thermal spring water.

Results: Clinical improvement was observed as early as Day 30, with a significant reduction in the total lesion count (from 401.75 to 322.25; p < 0.05), comedones (from 378.75 to 315.75; p < 0.05), and inflammatory lesions (from 23.25 to 6.75; p < 0.05). When excluding the nasal dorsum, the reduction was even more pronounced: total lesions decreased from 65 to 24.88; non-inflammatory lesions from 40.13 to 18.88; and inflammatory lesions from 21.63 to 6.25 (all p < 0.05). GEA scores progressively improved, with 25% of subjects lesion-free at D30. The mean mCADI score decreased from 6.25 to 5.13. The product was well tolerated with no treatment discontinuation.

Conclusions*:* The investigational topical product containing 7% Silybum marianum fruit extract, 0.1% retinaldehyde, 6% glycolic acid, and thermal spring water proved to be effective, safe, and well tolerated after 30 days of treatment in adults with acne.

## Introduction

Acne vulgaris is a common chronic inflammatory skin condition affecting individuals across various age groups. Although it is most prevalent during adolescence, acne can persist in adulthood or first appear after age 25. It is classified as adolescent acne (ages 10-19) and adult acne (≥25 years), with the latter subdivided into persistent, late-onset, and recurrent types. Persistent acne is the most common form, accounting for up to 82% of cases [1-4].

The pathogenesis of acne is complex and multifactorial, involving increased sebum production, follicular hyperkeratinization, inflammation, and colonization by *Cutibacterium acnes (C. acnes)*. Changes in sebum composition, such as variations in levels of squalene, triglycerides, and wax/cholesterol esters, also play a significant role. Although *C. acnes* is not hypercolonized in acne-prone skin, phylotype differences, particularly strains IA and IC, are observed between healthy individuals and those with acne-prone skin [5-7].Hormonal factors, diet, stress, smoking, genetic predisposition, and use of comedogenic cosmetics can contribute to the development and exacerbation of acne.

In addition to visible lesions, acne-prone skin presents subclinical abnormalities known as microcomedones, which are the initial stage of clinical lesions and create an environment favorable to *C. acnes*. Microcomedone formation results from dysregulated differentiation of sebaceous stem cells in the isthmus epithelium. Under normal conditions, these cells generate both the sebaceous gland and the epithelium of the sebaceous duct and isthmus [5,8,9]. In acne-prone skin, the loss of homeostasis leads to epithelial hyperproliferation and microcomedone formation. Thus, preserving the homeostasis of sebaceous stem cells has emerged as a novel therapeutic target in acne treatment.

Among innovative topical treatments, the *Silybum marianum* fruit (SMFE) bioactive extract has the potential to regulate keratinocyte differentiation and modulate comedone formation [10,11]. In addition to SMFE, other active agents have demonstrated effectiveness in modulating keratinization, reducing sebum production, and exerting antimicrobial and anti-inflammatory effects [11].

Retinaldehyde, a precursor of retinoic acid, promotes cell renewal and has antimicrobial activity against *C. acnes*. Topical retinoids are a mainstay in acne treatment due to their comedolytic and keratolytic effects. They also help reduce hyperpigmentation and scarring, promote papillary dermis regeneration, and have a pro-collagenase effect, making them a preferred option in acne cases with scarring or pigmentation concerns. Glycolic acid, an alpha-hydroxy acid, enhances epidermal exfoliation and improves skin texture [12]. Thermal spring water, rich in minerals and trace elements, has been associated with soothing and anti-inflammatory effects, enhancing skin tolerability to treatment [10,12].

Considering the need for effective and innovative therapeutic approaches, this pilot study aimed to evaluate the efficacy, safety, and tolerability of a topical formulation containing 7% Silybum marianum fruit extract, 0.1% retinaldehyde, 6% glycolic acid, and thermal spring water for the treatment of facial acne. We hypothesized that this combination would reduce both inflammatory and non-inflammatory acne lesions, while being well-tolerated and safe for daily use.

## Materials and methods

This single-center, open-label, prospective, single-arm pilot study was conducted at the Nomina Expert Institute in São Paulo, Brazil, in accordance with the Declaration of Helsinki and Good Clinical Practice principles. The study protocol was approved by the Institutional Review Board (approval number: 86111225.5.0000.5479). Written informed consent was obtained from all participants before enrollment. The study was not registered in a clinical trial registry, as it was designed and conducted as a pilot study with a limited number of participants (n=8). The authors considered registration optional.

Participants were recruited between May 5, 2025, and May 9, 2025. They were evaluated after 30 (±1) days at the second visit, which took place between June 4, 2025, and June 9, 2025. Eligible participants included men and women aged 19 to 25 with Fitzpatrick skin types I to VI, presenting with facial non-inflammatory acne, mild inflammatory acne, or moderate inflammatory acne. Exclusion criteria included pregnancy or lactation, ongoing acne treatments within the previous three months, or use of topical or systemic corticosteroids or immunosuppressants.

Clinical, efficacy, tolerability, and safety assessments

At baseline (D0), participants received the investigational product (IP) containing 7% *Silybum marianum* fruit extract (SMFE; Comedoclastin®), 0.1% retinaldehyde, 6% glycolic acid, and Avène thermal spring water, formulated as Cleanance Comedomed Peeling®. In addition, standardized cleansing gel (Cleanance Facial Cleansing Gel®) and sunscreen (Avène Mat Perfect Cleanance UV Defense SPF 70®) were provided for daily use over 30 days (D30).

Participants were instructed to apply a 30-day almond-sized amount of the IP to the entire face (excluding the eyes, eyelids, and lips) twice daily, in the morning and evening, after cleansing with the standardized facial cleanser. They were also advised to apply sunscreen after the morning application of the IP and to reapply it in the afternoon.

At baseline and after 30 days, subjects completed a medical history questionnaire and underwent a clinical evaluation by a dermatologist. Assessments included the Global Acne Severity Scale (GEA Scale) [13], which classifies acne as follows: 0 = clear skin, 1 = almost clear, 2 = mild, 3 = moderate, 4 = severe, and 5 = very severe. Volunteers also completed subjective evaluations regarding sebum production and the presence of dilated pores. Lesion counting was performed using Lucky's method [14], with facial regions recorded as the forehead, cheeks, nasal dorsum, and chin. Based on the lesion count results, the authors decided to present analyses that included and excluded the nasal dorsum, as the data from this area showed distinct patterns requiring specific interpretation.

The quality of life of patients with acne was assessed using the Cardiff Acne Disability Index (CADI) [15,16], which is designed for use in teenagers and young adults, at the two visits. The complete version consists of five questions; we used only four questions, as one of them refers to acne on the trunk, which did not meet the inclusion criteria. The tool was referred to as the modified CADI (mCADI) [17]. The questions offered responses that scored from 0 to 3. The higher the score, the greater the disease's impact on the patient's quality of life. In the complete questionnaire (five questions), total scores range from 0 to 15, with scores of 0-5 indicating a slight impact on quality of life, 6-10 indicating a moderate impact, and 11-15 suggesting a severe impact. A proportional adaptation was made: the total score per patient ranged from 0 to 12, with scores of (0;4) indicating a slight impact, (4;8) a moderate impact, and (8;12) a severe impact on quality of life. After 30 days, volunteers answered a subjective efficacy questionnaire and were also evaluated by dermatologists for assessments of tolerance and adverse effects. All individuals were photographed using a digital camera and the VECTRA software system (Canfield Scientific, Inc.) at baseline (D0) and after 30 days (D30).

Study objective

The primary objective was to evaluate the cutaneous acceptability, safety, and efficacy of a topical product containing 7% *Silybum marianum *fruit extract (Comedoclastin®), 0.1% retinaldehyde, 6% glycolic acid, and thermal spring water for the treatment of facial acne in adult patients for 30 days.

Statistical analysis

Lesion count data (total, excluding the nasal dorsum, non-inflammatory, and inflammatory) from Day 0 (D0) and Day 30 (D30) were analyzed using measures of central tendency, dispersion, and position. Confidence intervals were computed using Student's t-statistics for paired data, with a significance level of 5%, following confirmation of data normality, using the Shapiro-Wilk test. Subjective questionnaire responses were assessed using response frequency distributions across the study period. Outliers were evaluated using boxplot analysis. Statistical analyses were conducted using R v4.1.3 (R Foundation for Statistical Computing, Vienna, Austria), Excel, and Jamovi (open software).

## Results

Demographic characteristics

In this pilot study, we enrolled eight patients (four female patients and four male patients) aged between 19 and 25. Five participants (62.5%) were between 18 and 22 years old, and three (37.5%) were between 22 and 25. We recorded the following distribution of Fitzpatrick skin phototypes: Type I - 1 participant (12.5%), Type III - 2 (25.0%), Type IV - 4 (50.0%), and Type V - 1 (12.5%).

Efficacy

**Figure 1:**
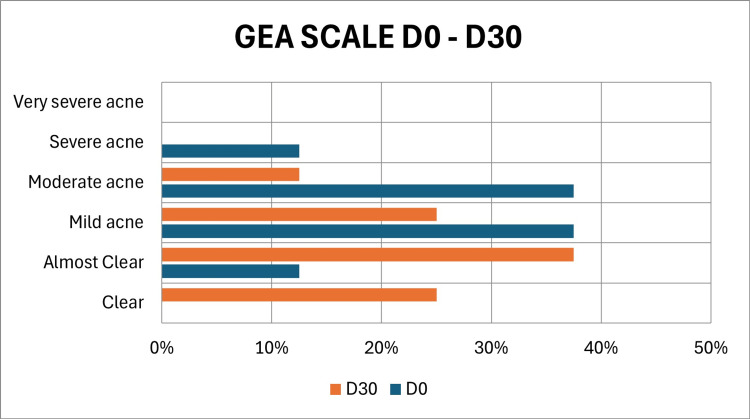
Progression of Global Acne Severity Scale (GEA) scores in subjects after 30 days of treatment with a topical formulation containing 7% Silybum marianum fruit extract (SMFE), 0.1% retinaldehyde, 6% glycolic acid, and thermal water.

At baseline (D0), according to the Global Acne Severity Scale (GEA), one volunteer (12.5%) presented with almost clear skin (GEA 1), 3 (37.5%) with mild acne (GEA 2), 3 (37.5%) with moderate acne (GEA 3), and 1 (12.5%) with severe acne (GEA 4). By Day 30 (D30), two volunteers (25%) had no visible acne lesions (GEA 0), three volunteers (37.5%) were classified as almost clear (GEA 1), two volunteers (25.0%) had mild acne (GEA 2), and one volunteer (12.5%) remained with moderate acne (Figure 1).

**Figure 2:**
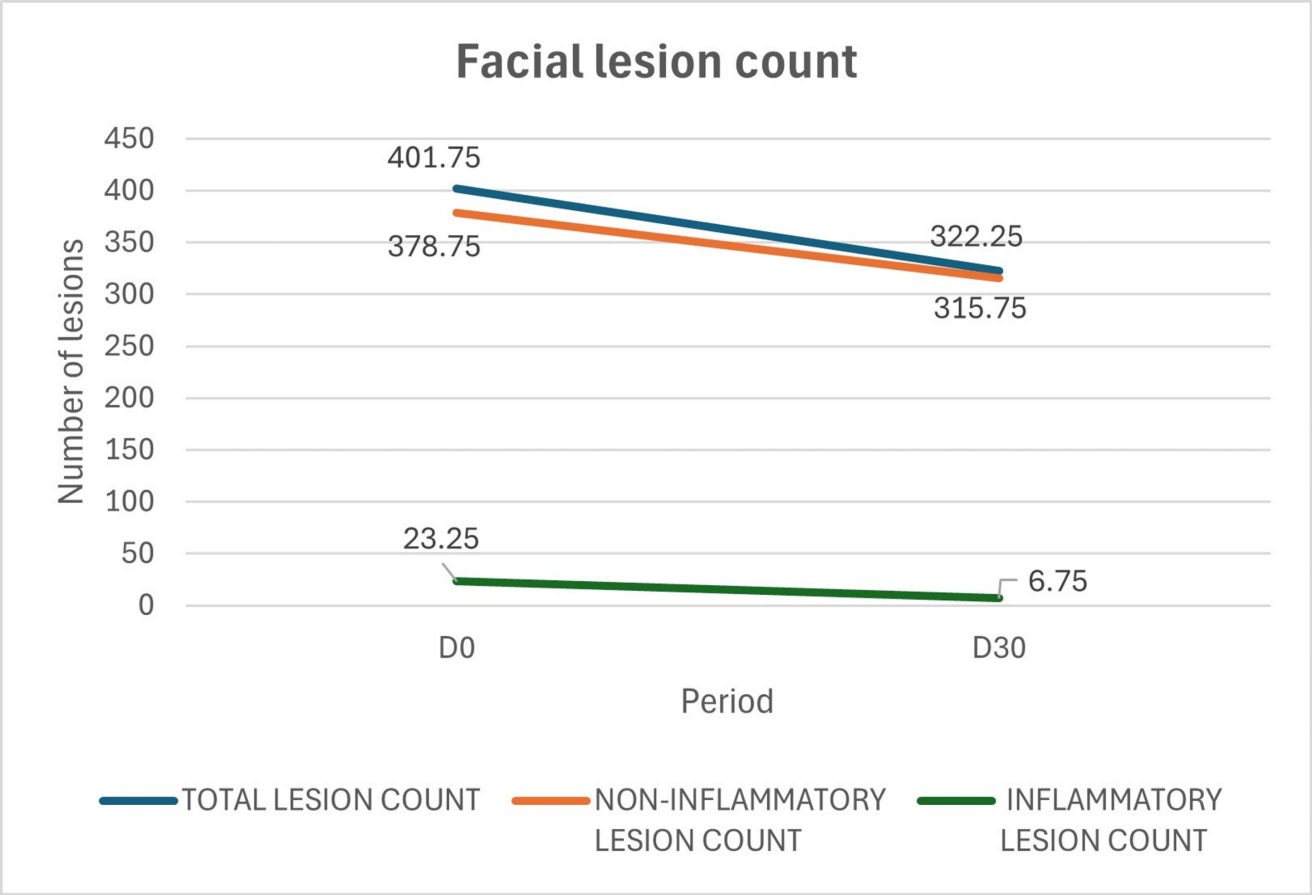
Clinical evolution of inflammatory and non-inflammatory facial acne lesions after treatment with the investigational product containing Silybum marianum fruit extract (SMFE), 0.1% retinaldehyde, 6% glycolic acid, and thermal water. The facial total lesion count decreased by 19.79%. Non-inflammatory lesions (open and closed comedones) across the face decreased by 16.63%. Inflammatory lesions (papules, pustules, nodules) declined by 70.97%.

Using Lucky's method, the facial total lesion count decreased 19.79% from a mean of 401.75 at baseline to 322.25 at D30 (95% CI: −31.89% to −7.68%), paired t-test: p=0.009, critical t-value=2.3646, t statistic=3.611. Non-inflammatory lesions (open and closed comedones) across the whole face decreased 16.63% from 378.75 to 315.75 (95% CI: −29.94% to −3.32%), paired t-test: p=0.026, critical t-value=2.3646, t statistic=2.803. Inflammatory lesions (papules, pustules, nodules) decreased 70.97%, with a mean reduction from 23.25 to 6.75 (95% CI: −98.33% to −43.60%), paired t-test: p=0.016, critical t-value=2.3646, and t statistic=3.144. All the results showed statistically significant reduction, as shown in Table 1 and Figure 2.

**Table 1:** Study population characteristics and clinical outcomes of eight volunteers using a cream combining 7% Silybum marianum fruit extract, 0.1% retinaldehyde, and 6% glycolic acid, along with thermal water.

Gender			n (%)	
	Female		4 (50.00)	
	Male		4 (50.00)	
	Total		8 (100.00)	
Age Group (Years)	0-18		0 (0.00)	
	18-22		5 (62.50)	
	22-26		3 (37.50)	
	Total		8 (100.00)	
Fitzpatrick Skin Types	I		1 (12.50)	
	II		0 (0.00)	
	III		2 (25.00)	
	IV		4 (50.00)	
	V		1 (12.50)	
	VI		0 (0.00)	
	Total		8 (100.00)	
GEA Scale			n (%)	
			D0	D30
					0	Clear. No lesions	0 (0.00)	2 (25.00)
	1	Almost clear. Almost no lesions	1 (12.50)	3 (37.50)				
					2	Mild acne	3 (37.50)	2 (25.00)
	3	Moderate acne	3 (37.50)	1 (12.50)
	4	Severe acne	1 (12.50)	0 (0.00)
					5	Very severe acne	0 (0.00)	0 (0.00)
	Total		8 (100.00)	8 (100.00)				
	Total Lesion Count			D0	D30
		Mean		401.75	322.25
		Standard Deviation		175.84	200.75
		Coefficient of Variation		43.77%	62.30%
		Standard Error		62.17	70.98
Total Number of Lesions, Excluding Nasal Dorsum			D0	D30
	Mean		65	24.88
	Standard Deviation		39.32	15.5
	Coefficient of Variation		60.49%	62.30%
	Standard Error		13.9	5.48
Non-inflammatory Lesion Count			D0	D30
	Mean		378.75	315.75
	Standard Deviation		168.75	200.56
	Coefficient of Variation		44.56%	63.52%
	Standard Error		59.66	70.91
Non-inflammatory Lesion Count, Excluding Nasal Dorsum			D0	D30
	Mean		40.125	18.88
					Standard Deviation		22.9	13.67
	Coefficient of Variation		57.07%	72.45%
	Standard Error		8.1	4.83
	Total Inflammatory Lesion Count			D0	D30
Mean		23.25	6.75
Standard Deviation		19.29	5.52
Coefficient of Variation		82.98%	81.82%
Standard Error		6.82	1.95
Inflammatory Lesion Count, Excluding Nasal Dorsum			D0	D30
					Mean		21.625	6.25
	Standard Deviation		16.18	4.68
	Coefficient of Variation		74.81%	74.92%
	Standard Error		5.72	1.66
	m CADI			SCORE	
Estimador\Di		D0	D30
Mean		6.25	5.125
Standard Deviation		3.012	1.246
Standard Error		1.064	0.44
Confidence Interval 95% (z)		[3.732;	[4.083;
		8.768	6.167]
Minimal punctuation		3	3
Maximal punctuation		11	7
Effectiveness of the Treatment for Oiliness			D30	
	No efficacy		0 (0.0%)	
	Low efficacy		0 (0.0%)	
	Moderate efficacy		2 (25.0%)	
	Good efficacy		3 (37.50%)	
	Excellent efficacy		3 (37.50%)	
Efficacy of the Treatment in Controlling New Acne Lesions			D30	
	No efficacy		0 (0.00%)	
					Low efficacy		0 (0.00%)	
	Moderate efficacy		1 (12.50%)					
					Good efficacy		4 (50.00%)	
	Excellent efficacy		3 (37.50%)					
								D30	
	Tolerability of the Treatment	Very low tolerance to product use, with strong discomfort and objective signs requiring discontinuation of daily care with the product.		0 (0.00%)	
		Low tolerance to product use, with complaints or objective signs requiring discontinuation of daily care with the product.		0 (0.00%)	
		Good tolerance, with minimal and temporary discomfort not requiring discontinuation of daily care with the product.		4 (50.00%)	
		Good tolerance with no objective signs observed upon examination.		1 (12.50%)	
Very good tolerance, with no complaints of discomfort and no objective signs identified upon examination.		3 (37.50%)	

**Figure 3:**
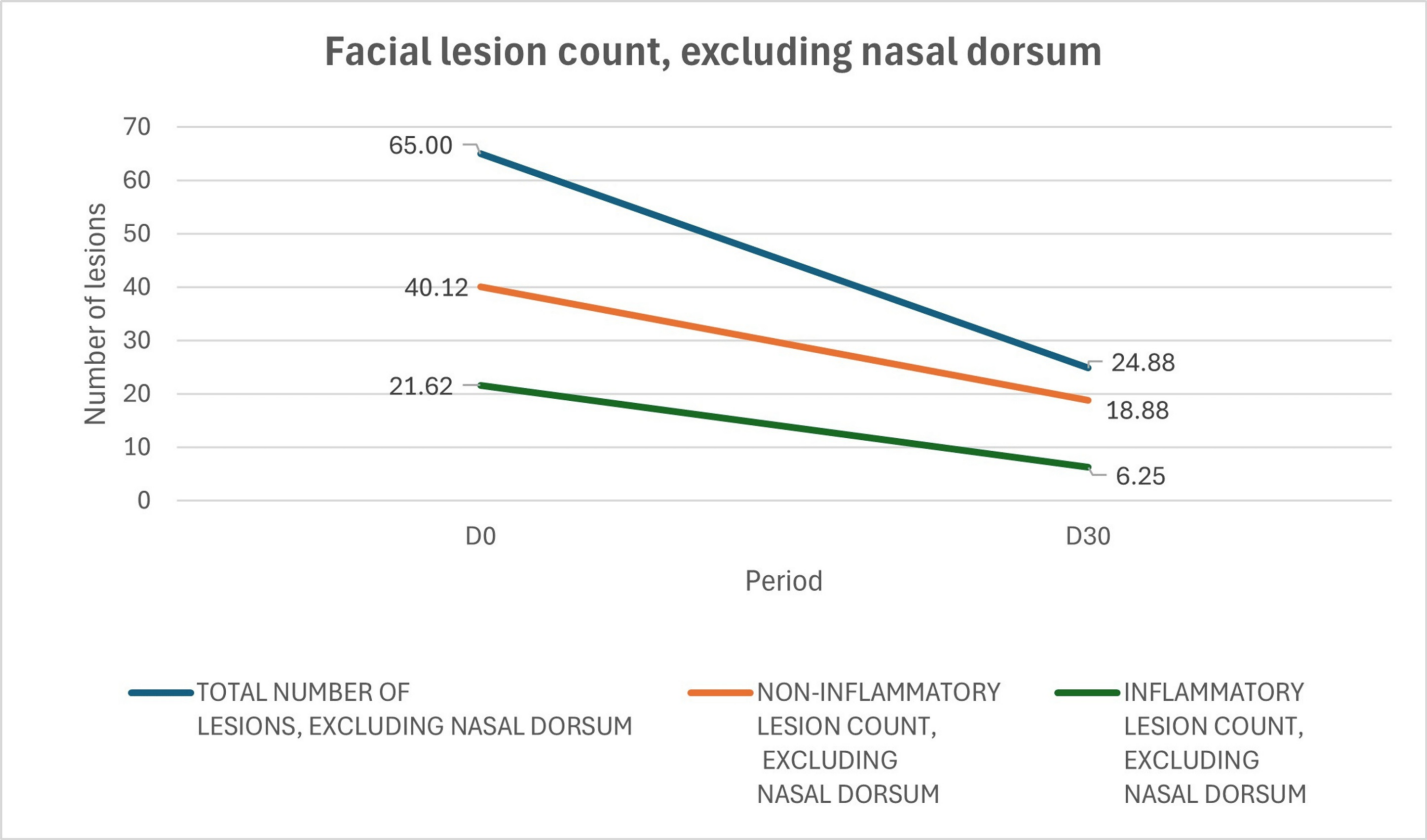
Clinical evolution of inflammatory and non-inflammatory facial acne lesions excluding nasal dorsum after treatment with the investigational product containing Silybum marianum fruit extract (SMFE), 0.1% retinaldehyde, 6% glycolic acid, and thermal water. The mean total lesion count excluding the nasal dorsum decreased by 61.73%; non-inflammatory lesions without nasal dorsum showed a reduction of 52.96%, and inflammatory lesions without nasal dorsum decreased by 71.10%.

After interpreting the results, the authors decided to also describe data without the nasal dorsal area, given its high concentration of comedones and distinct behavior; lesion reduction was even more pronounced and statistically significant when the nasal dorsum was excluded from the analysis. Mean total lesions without nasal dorsum decreased 61.73% from 65.00 to 24.88 (95% CI: −72.14% to −51.32%), paired t-test: p=0.004, critical t-value=2.3646, t statistic=4.134; non-inflammatory lesions without nasal dorsum showed reduction of 52.96% from 40.13 to 18.88 (95% CI: −69.38% to −36.53%), paired t-test: p=0.004, critical t-value=2.3646, t statistic=4.209; and 71.10% of reduction of inflammatory lesions without nasal dorsum from 21.63 to 6.25 (95% CI: −94.39% to −47.80%), paired t-test: p=0.011, critical t-value=2.3646, t statistic=3.438 (Figure 3).

According to the subjective efficacy questionnaire regarding oiliness control after 30 days, three participants (37.5%) rated the efficacy as excellent, three (37.5%) as very good, and two (25%) as moderate. In terms of controlling new acne lesions, three participants (37.5%) rated the result as excellent, four (50%) as very good, and one (12.5%) as moderate (Table 1).

**Figure 4:**
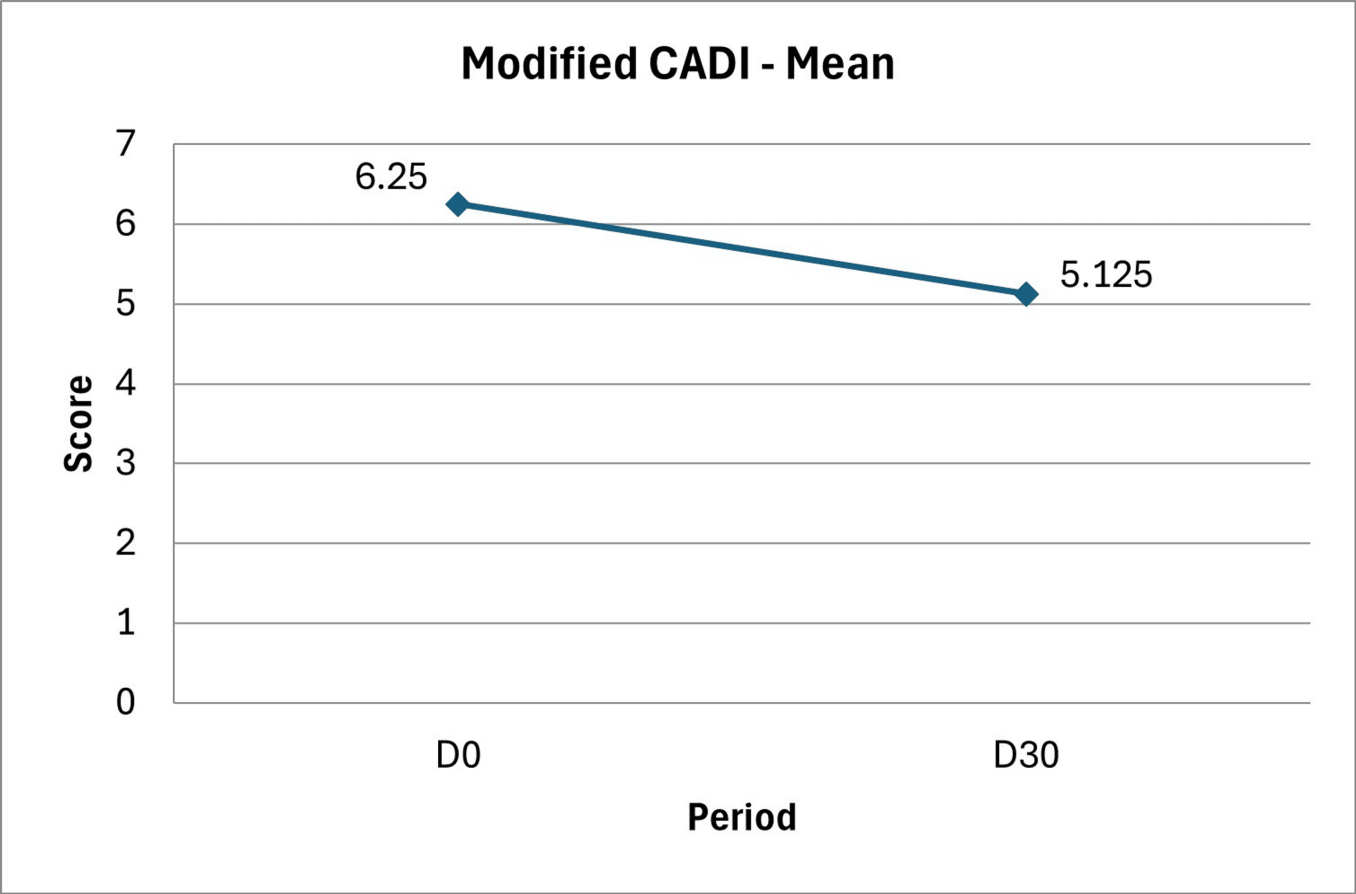
Modified CADI (Cardiff Acne Disability Index) in eight volunteers who used 30 days of topical treatment with the investigational product containing 7% Silybum marianum fruit extract, 0.1% retinaldehyde, 6% glycolic acid, and thermal water.

Regarding quality of life, participants answered the modified CADI (mCADI). When asked about feelings of irritation, embarrassment, or shame caused by facial acne and oily skin, only one participant (12.5%) reported no such issues before treatment. In contrast, four participants (50.0%) denied those complaints after 30 days of use. At D0, four participants (50.0%) reported being highly bothered, compared to only one (12.5%) at D30. The mean mCADI score decreased 18%, from 6.25 (95% CI: 3.73 to 8.77) to 5.13 (95% CI: 4.08 to 6.17), paired t-test: p=0.4113, critical t-value=2.3646, t statistic=0.8736 (Figure 4).

**Figure 5:**
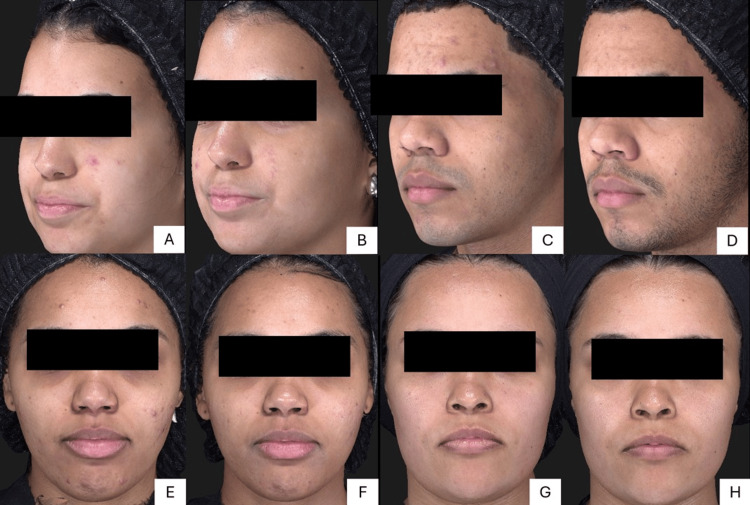
Clinical aspect of acne vulgaris before (A,C,E,G), and after (B,D,F,H) treatment. Representative images showing the differences between acne vulgaris before and after treatment with topical therapy with the investigational product containing 7% Silybum marianum fruit extract, 0.1% retinaldehyde, 6% glycolic acid, and thermal water for 30 days. Written informed consent for publication in an open-access journal was obtained from the volunteers.

In this pilot study, the authors evaluated volunteers' photographs, and improvements were observed in inflammatory and non-inflammatory lesions and skin quality, as described in Figure 5.

Safety and tolerability

Dermatologists evaluated tolerability. Four participants (50.0%) experienced minimal and transient discomfort that did not require treatment interruption. One (12.5%) showed good tolerability without objective dermatologic signs, and three (37.5%) showed excellent tolerability without discomfort or objective findings.

Only one adverse event was reported: one participant experienced stinging on the first day of product application, which resolved spontaneously and required no change in instructions.

## Discussion

The inhibition of microcomedone formation and the consequent reduction of inflammatory and non-inflammatory lesions remains a significant challenge in acne management. SMFE acts on the comedone switch mechanism, protecting sebocytes from potent comedogenic agents such as 2,3,7,8-tetrachlorodibenzo-p-dioxin (TCDD) and modulating the expression of keratins (K75 and K79) and lipid drop proteins: perilipin 2 protein (Plin2) and CIDEA protein (CIDEA) involved in sebaceous stem cell lineages [10,11].

Clinically, topical SMFE treatment has demonstrated successful outcomes in more than 84% of cases in a study involving over 4,000 patients [18]. Reflectance confocal microscopy has been shown to be efficacious in reducing the diameter of follicular infundibula and microcomedones [17]. Additionally, repeated skin surface biopsies have supported the reduction in microcomedone index, showing maintenance of lower lesion counts over time [11].

Retinaldehyde is well known for its comedolytic activity [19] and its additional antibacterial effect against gram-positive bacteria, including C. acnes [20] due to its aldehyde group. A cosmetic formulation containing 0.1% retinaldehyde and 6% glycolic acid has been shown to be well tolerated and superior to vehicle in patients with mild to moderate acne [21]. Alpha-hydroxy acids, particularly glycolic acid, exhibit keratolytic properties at 5-10% concentrations. The association of these established actives with SMFE produced effective clinical results after 30 days of treatment in our pilot study involving patients with mild to moderate inflammatory acne.

Improved GEA scores (Figure 1) and lesion counts via Lucky's method (Table 1) documented clinical perception of reduced inflammatory lesions. The detailed analysis included full-face data and a region-specific evaluation excluding the nasal dorsum, which typically presents a much higher density of comedones. This sectoral analysis enabled a clearer understanding of response patterns across different facial zones (Figures 2, 3), showing statistically significant reductions. Additionally, after 30 days, most participants considered the product to be excellent or very good in terms of reducing oiliness and controlling new acne lesions.

Figure 4 illustrates the improvement in quality of life as assessed by the mCADI, showing better outcomes in the psychological and social domains (Questions 1 and 2) and in personal perception of acne severity (Questions 3 and 4) [16]. Although an 18% reduction was observed, the difference was not statistically significant.

Pre- and post-treatment photographs (Figure 5) demonstrate improved inflammatory lesions and skin quality. Subjective efficacy and tolerability questionnaires confirmed the investigational product's safety profile.

Limitations

This was a non-controlled pilot study. To confirm these preliminary findings and allow for more robust comparisons, a future trial with a larger sample size and an active comparator aligned with current acne treatment guidelines is needed.

## Conclusions

In this single-arm pilot study, the investigational topical product, containing 7% Silybum marianum fruit extract, 0.1% retinaldehyde, 6% glycolic acid, and thermal spring water, demonstrated clinically meaningful results. After 30 days of use, participants experienced reductions in both inflammatory and non-inflammatory facial lesions, with statistically significant improvements, and reported high levels of patient satisfaction regarding the control of oiliness and lesion reduction. The product also demonstrated good tolerability and safety, with no treatment discontinuations, as well as improvements in skin quality. Despite not being statistically significant, there was an improvement in the quality-of-life score.

These findings suggest that this combination may represent a promising option for managing mild to moderate acne, particularly in patients seeking well-tolerated treatments with anti-inflammatory and comedolytic action. Further randomized studies are needed to confirm these results and explore long-term outcomes.

## Appendices

Anamnesis

VISIT: D0 Date of visit: ................./................./..................... Volunteer Information: Initials: ................................................................................ Gender: F ( ) M ( ) Date of birth: ...................................................................................... Medical History: Concomitant diseases: Yes ( ) No ( ) If yes, which ones? ..................................................................................... Allergies: Yes ( ) No ( ) If yes, which ones? ..................................................................................... Current medication use at the time of this visit: Yes ( ) No ( ) If yes, which ones? ..................................................................................... Disease History: Onset of acne: ..................................................................................... Family history of acne: Yes ( ) ...................................................... No ( ) Previous acne treatments: Yes ( ) No ( ) If yes, which ones? ( ) Facial hygiene products (dermocosmetics): .............................................. ( ) Topical anti-acne dermocosmetic products: .............................................. ( ) Topical medications: ............................................................................... ( ) Systemic medications: ................................................................................ Current acne treatments: Yes ( ) No ( ) If yes, which ones? ( ) Facial hygiene products (dermocosmetics): ..............................................

( ) Topical anti-acne dermocosmetic products: .............................................. ( ) Topical medications: ............................................................................... ( ) Systemic medications: ................................................................................ ( ) Use of oral contraceptives: Yes ( ) No ( ) Which one? .................. For how long? ............. Date of last menstruation: .................. ( ) Use of hormonal IUD? Yes ( ) No ( )

Subjective efficacy assessment questionnaire after 30 days of product use

Date of visit: ................./................./..................... Volunteer Information: Initials: ................................................................................ Gender: F ( ) M ( ) 1. Considering the results observed after 30 days of using this product, how would you rate its efficacy in controlling your skin’s oiliness? 0 - No efficacy 1 - Low efficacy 2 - Moderate efficacy 3 - Good efficacy 4 - Excellent efficacy 2. Considering the results observed after 30 days, how would you rate this product’s efficacy in preventing the appearance of new acne lesions on a 5-point scale? 0 - No efficacy 1 - Low efficacy 2 - Moderate efficacy 3 - Good efficacy 4 - Excellent efficacy
